# Identification of horse chestnut coat color genotype using SNaPshot^®^

**DOI:** 10.1186/1756-0500-2-255

**Published:** 2009-12-16

**Authors:** Fernando Rendo, Mikel Iriondo, Carmen Manzano, Andone Estonba

**Affiliations:** 1 Department of Genetics, Physical Anthropology and Animal Physiology, Faculty of Science and Technology, University of the Basque Country, E-48940 Bilbao, Spain

## Abstract

**Background:**

The Cantabrian Coast horse breeds of the Iberian Peninsula have mainly black or bay colored coats, but alleles responsible for a chestnut coat color run in these breeds and occasionally, chestnut horses are born. Chestnut coat color is caused by two recessive alleles, *e *and *e*^*a*^, of the melanocortin-1 receptor gene, whereas the presence of the dominant, wild-type *E *allele produces black or bay coat horses. Because black or bay colored coats are considered as the purebred phenotype for most of the breeds from this region, it is important to have a fast and reliable method to detect alleles causing chestnut coat color in horses.

**Findings:**

In order to assess coat color genotype in reproductive animals with a view to avoiding those bearing chestnut alleles, we have developed a reliable, fast and cost-effective screening device which involves Single Nucleotide Polymorphism (SNP) detection based on SNaPshot^® ^(Applied Biosystems) methodology. We have applied this method to four native breeds from the Iberian Cantabrian Coast: Pottoka and Jaca Navarra pony breeds, in which only black or bay coats are acceptable, and Euskal Herriko Mendiko Zaldia and Burguete heavy breeds, in which chestnut coats are acceptable. The frequency of the chestnut alleles ranged between *f *= 0.156-0.322 in pony breeds and between *f *= 0.604-0.716 in heavy breeds.

**Conclusions:**

This study demonstrates the usefulness of the DNA methodology reported herein as a device for identifying chestnut alleles; the methodology constitutes a valuable tool for breeders to decrease the incidence of chestnut animals among Cantabrian Coast pony breeds.

## Findings

Local horse breeds in the Cantabrian Coast of the Iberian Peninsula include the Pottoka, Jaca Navarra, Losino, Monchino, Asturcón, Galego and Garrano ponies, and the Euskal Herriko Mendiko Zaldia (*EHMZ*) and Burguete heavy breeds. With a view to preserving these breeds, purebred standards have recently been defined. For the pony breeds, black and bay colors are considered to be the original coat colors [1,2], even though chestnut individuals do exist, while both bay and chestnut coats are the typical coat colors in the *EHMZ *and Burguete breeds. The absence of chestnut horses in the Iberian Peninsula until the Medieval Age [3], together with the naturally occurring isolation of these breeds, suggest that introgression of chestnut alleles most likely occurred around the 1930s when local mares were crossed with stallions of Breton, Percheron and Ardennais breeds, which are mainly chestnut, to improve the capacity for agricultural work and meat production [4]. For this reason, chestnut coat color individuals are not allowed to be included in the official stud book in the Cantabrian Coast pony breeds. In order to facilitate the selection and enrichment of the purebred populations of these horses, in terms of coat color, we propose a method to assess the coat color genotype among reproductive animals, since chestnut color is known to be a genetically recessive trait.

Chestnut coat color is controlled by the *Extension *gene [5], which encodes the melanocortin-1 receptor (*MC1R*). In horses, three alleles responsible for the major coat phenotypes have been identified in the *MC1R *exon 1. Marklund *et al*. [6] identified a chestnut *e *allele resulting from a single missense mutation at position C901T (S83F). Later, Wagner and Reissmann [7] identified a second chestnut allele *e*^*a*^, resulting from another single missense mutation at position G903A (D84N). The wild-type allele *E *is dominant at the *MC1R locus*, and its presence produces a black or bay coat, whereas the recessive *e *and *e*^*a *^alleles give rise to a chestnut coat when they are present in a homozygous or heterozygous configuration [7].

The design of a simple method for the quick identification of mutations responsible for chestnut coat color is of interest to many horses breeders. This identification is usually performed using the Polymerase Chain Reaction based on Restriction Fragment Length Polymorphisms (PCR-RFLP) [6], which is a time-consuming technique involving a high level of manipulation. Also an RT-PCR based method has recently been proposed [8]. These two methodologies are not a cost-effective techniques when additional single nucleotide polymorphism (SNP)-based markers need to be analyzed simultaneously. Here, we describe a fast, cost-effective and reliable alternative method for the routine genotyping of alleles causing chestnut coat color in horses based on SNaPshot^®^. This SNP detection methodology involves few manipulation steps, all of which are susceptible to automation, and has scalable flexibility for low- and high-throughput capacity [9].

## Materials and methods

**Figure 1:**
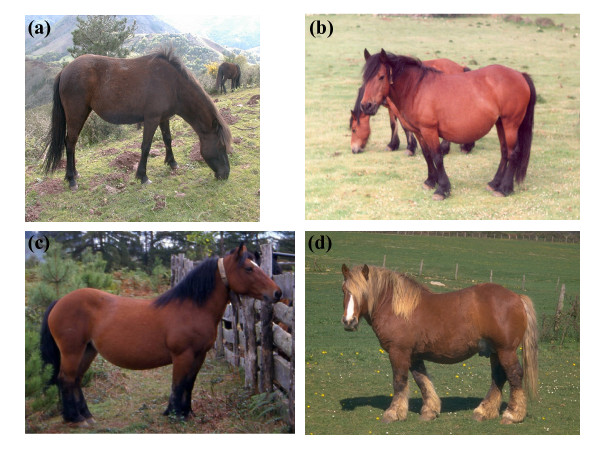
**The four native breeds analyzed**. (a) Pottoka and (b) Jaca Navarra ponies; (c) Euskal Herriko Mendiko Zaldia and (d) Burguete heavy horse breeds.

125 Pottoka, 56 Jaca Navarra, 24 *EHMZ *and 44 Burguete purebred horses from the Western Pyrenees (northern Spain) were sampled, all of them included in their respective stud books (Figure 1). Genomic DNA was isolated from blood using a STARlet automatic robot (Hamilton) and DNeasy 96 Blood & Tissue Kit (Qiagen). SNaPshot^® ^performs an SNP genotyping assay based on a single-base extension method. First, a 154-bp fragment of *MC1R *exon 1 was amplified from genomic DNA using 5'-GCAACCTGCACTCACCCAT-3' (forward) and 5'-TTGTCCAGCTGCTGCAACA-3' (reverse) primers. All PCR reactions were carried out in 5 μl volumes with 5-20 ng template DNA, 0.2 mM dNTPs, 0.3 μM of each primer and 1.0 U of AmpliTaq Gold polymerase in the accessory Buffer II (Applied Biosystems) and with 1.5 mM MgCl_2_. A PCR program consisting of initial denaturation at 95°C for 10 min, 35 cycles at 95°C for 20 s, 57°C for 20 s, and 72°C for 30 s, and a final extension at 72°C for 10 min was performed in a Veriti™ 96-Well Thermal Cycler (Applied Biosystems). All PCR products were cleaned up with 1.5 μl of ExoSAP-IT^® ^(USB Corporation) at 37°C for 45 min and then incubated at 80°C for 15 min. PCR products were then used to perform the specific SNaPshot^® ^reaction containing C901T SNP-specific primer (5'-ATATTGCTGCCTGGCCGTGT-3') and G903A SNP-specific primer (5'-ATATATTGCTCATGCTCACCAGCAGGT-3'). All SNaPshot^® ^reactions were carried out in a volume of 6 μl which included 1.8 μl of cleaned up PCR products, 0.2 μM of each specific primer and 0.85 μl of SNaPshot^® ^reagents, with a program including an initial denaturation at 96°C for 1 min and 25 cycles consisting of 96°C for 10 s, 60°C for 35 s, in the same thermal cycler used before. All SNaPshot^® ^specific products were cleaned up with 1 μl of Shrimp Alkaline Phosphatase - SAP (Promega Biotech) at 37°C for 60 min and then incubated at 75°C for 15 min. Finally, these SNaPshot^® ^specific products were analyzed by capillary electrophoresis in a 3130xl Genetic Analyzer and with GeneMapper Software (Applied Biosystems) according to manufacturer's instructions. Hardy-Weinberg equilibrium (HWE) tests and Expected Heterozygosity (H_e_) were calculated using GENEPOP software, version 4.0 [10].

## Results and Discussion

**Figure 2:**
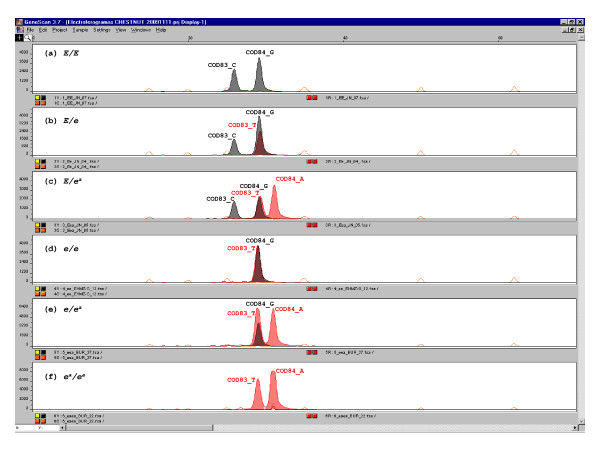
**Electropherograms for the different genotypes**. (a) *E/E *(C/C; G/G), (b) *E/e *(C/T;G/G), (c) *E/e*^*a *^(C/T;G/A), (d) *e*/*e *(TT; GG), (e) *e*/*e*^*a *^(TT; GA) and (f) *e*^*a*^/*e*^*a *^(TT; AA).

Electropherograms of the 249 analyzed samples exhibited the six different possible genotypes: *E/E*, *E/e*, *E/e*^*a*^, *e/e*, *e/e*^*a *^and *e*^*a*^/*e*^*a *^(Figure 2). A sequencing assay was carried out for at least two individuals of each genotype, except for the *e*^*a*^/*e*^*a *^genotype, for whom only one individual was identified, using the Big-Dye-Terminator v3.1 protocol (Applied Biosystems) in a 3130xl Genetic Analyzer, in order to validate the "chestnut coat-color" genotypes and verify that the electropherograms corresponded to the expected sequences. In all cases analyzed, the results obtained were successful. Genotypes and allele frequencies are shown in Table 1. Pottoka, *EHMZ *and Burguete samples are in HWE, whereas Jaca Navarra sample is not. This may be due to the deliberate exclusion practiced by Jaca Navarra breeders of chestnut individuals, who are not accepted in the stud book. For this reason, allele frequencies for this breed should be interpreted with caution.

**Table 1:** Sample size (N), genotypes and frequencies of the *E*, *e *and *e*^*a *^alleles of the *Extension *gene and Hardy-Weinberg Equilibrium (HWE) test results for the Pottoka and Jaca Navarra ponies and for the Euskal Herriko Mendiko Zaldia (EHMZ) and Burguete heavy-horse breeds.

Breed	Pottoka	Jaca Navarra	EHMZ	Burguete
N	125	56	24	44
*Genotypes*				
*E/E*	86	20	2	4
*E/e*	38	34	11	17
*E/e*^*a*^	1	2	4	0
*e/e*	0	0	7	18
*e/e*^*a*^	0	0	0	4
*e*^*a*^/*e*^*a*^	0	0	0	1
*Allele frequencies*				
*E*	0.844 ± 0.023	0.679 ± 0.044	0.396 ± 0.071	0.284 ± 0.048
*e*	0.152 ± 0.023	0.304 ± 0.043	0.521 ± 0.072	0.648 ± 0.051
*e*^*a*^	0.004 ± 0.004	0.018 ± 0.013	0.083 ± 0.040	0.068 ± 0.027
HWE *p *value	0.1347 ± 0.0004	0.0007 ± 0.0000	0.0739 ± 0.0002	0.1604 ± 0.0002

The results obtained reveal two important findings: firstly, the *e*^*a *^mutation seems to be present in several Cantabrian Coast horse breeds, since up to now, this mutation has only been reported to be present in the Asturcón pony breed [11]; secondly, the frequency of alleles causing chestnut coat color (*e *and *e*^*a*^) is highly variable in these breeds, ranging from *f *= 0.156 ± 0.023 in Pottoka to *f *= 0.716 ± 0.048 in Burguete. If we interpret these frequencies to be in part indicative of the degree of introgression of foreign genetic material into the breed, then the present results suggest an important degree of mixing in the pony breeds, especially in the Jaca Navarra breed. Since the aim of breeders of Cantabrian Coast ponies is to decrease the incidence of chestnut animals among these horses, these results underline the need for mating management which avoids reproductive animals who carry chestnut alleles; the DNA tools presented herein will be very useful for genotyping to this end. It should be stressed that if individuals who carry the *e *and *e*^*a *^alleles are excluded from the reproductive processes of the Pottoka and Jaca Navarra breeds (genotypes for the Agouti-signaling peptide gene are also used in Jaca Navarra [12]), there would not be a significant reduction of genetic variability in either of the two breeds. An analysis of these samples using 17 microsatellites (*unpublished results*), showed that the expected heterozygosity of the Jaca Navarra population would change from H_e _= 0.744 (N = 56) to H_e _= 0.751 (N_*E/E *_= 20), while in the case of Pottoka, this would change from H_e _= 0.779 (N = 125) to H_e _= 0.778 (N_*E/E *_= 86).

The method described herein is a fast, cost-effective and reliable alternative for the routine genotyping of alleles causing chestnut coat color in horses. We estimate a cost per SNP and per individual of 1.59 Euro when analyzing two SNPs (excluding cost associated with DNA extraction). This cost could be further reduced to 0.42 Euro per SNP, per individual when 10 SNPs are analyzed, or to 0.21 Euro when 25 SNPs are analyzed simultaneously [9]. In contrast, the RT-PCR based method [8] involves a similar expense for two SNPs, but this cost would increase in a manner approximately proportionate to the number of analyzed SNPs (Applied Biosystems, *pers. comm*.). The SNaPshot^® ^methodology which we propose herein is fast insofar as it is a medium-throughput technology. Moreover, it presents the additional advantage that increasing the number of analyzed SNPs only slightly increases the total time necessary to obtain results.

## Conclusions

In conclusion, we have developed and validated a SNaPshot^® ^based methodology for the robust, reliable and reproducible genotyping of chestnut coat color alleles in a fast, simple and cost-effective way. Furthermore, since SNaPshot^® ^can analyze more than 10 SNPs at the same time [9], this is not a closed method but an open one, where more markers for multiple desired traits can be added allowing their simultaneous routine genotyping. Finally, this methodology, proven in this study to be useful for four Cantabrian Coast horse breeds, it is not exclusive to these horses breeds; rather it could be extended to any horse breed and, therefore, to any breeders association, which needs to know the 'chestnut coat color' associated genotype for a certain reproductive animal.

## Competing interests

The authors declare that they have no competing interests.

## Authors' contributions

FR performed all SNaPshot^® ^methodology concerning analysis, DNA genotyping and sequencing, as well as drafted the manuscript. MI and CM participated in the design of the study as well as in manuscript writing and editing. AE conceived the study and was responsible for funding and supervising the research project. All authors approved the final manuscript.
